# The Less the Better: How Suppressed Base Addition Boosts Production of Monoclonal Antibodies With Chinese Hamster Ovary Cells

**DOI:** 10.3389/fbioe.2019.00076

**Published:** 2019-04-11

**Authors:** Max Becker, Lisa Junghans, Attila Teleki, Jan Bechmann, Ralf Takors

**Affiliations:** ^1^Institute of Biochemical Engineering, University of Stuttgart, Stuttgart, Germany; ^2^Boehringer Ingelheim Pharma GmbH & Co. KG, Biberach an der Riss, Germany

**Keywords:** CHO, fed-batch, CO_2_, pH, flux analysis, metabolomics, productivity, lactate

## Abstract

Biopharmaceutical production processes strive for the optimization of economic efficiency. Among others, the maximization of volumetric productivity is a key criterion. Typical parameters such as partial pressure of CO_2_ (pCO_2_) and pH are known to influence the performance although reasons are not yet fully elucidated. In this study the effects of pCO_2_ and pH shifts on the phenotypic performance were linked to metabolic and energetic changes. Short peak performance of q_mAb_ (23 pg/cell/day) was achieved by early pCO_2_ shifts up to 200 mbar but followed by declining intracellular ATP levels to 2.5 fmol/cell and 80% increase of q_Lac_. On the contrary, steadily rising q_mAb_ could be installed by slight pH down-shifts ensuring constant cell specific ATP production (q_ATP_) of 27 pmol/cell/day and high intracellular ATP levels of about 4 fmol/cell. As a result, maximum productivity was achieved combining highest q_mAb_ (20 pg/cell/day) with maximum cell density and no lactate formation. Our results indicate that the energy availability in form of intracellular ATP is crucial for maintaining antibody synthesis and reacts sensitive to pCO_2_ and pH-process parameters typically responsible for inhomogeneities after scaling up.

## Introduction

The global sales for biopharmaceuticals are constantly on the rise which is the major driving force for the optimization of industrial production processes (Walsh, 2014; Morrison and Lähteenmäki, 2017). Additionally, the growing number of expiring patent protections and the subsequent emergence of biosimilar producing companies and processes has increased the urge for optimized processes even further (Mullard, 2012; Gaughan, 2016). Especially the major biopharmaceutical product group of monoclonal antibodies and their main production host Chinese hamster ovary (CHO) cells are in the focus of research (Ecker et al., 2015). As a result, product titers have increased substantially over the past years (Kunert and Reinhart, 2016) and process intensification toward continuous processes (Hammerschmidt et al., 2014) as well as an increased use of single-use bioreactors (Löffelholz et al., 2013) has taken place. But since fed-batch cultivation remains the predominant large-scale process in industry, a large number of studies focusing on fed-batch processes have been published over the past decades. An improved understanding of media and feeding was therefore already achieved (Birch and Racher, 2006; Shukla and Thömmes, 2010). Despite the gathered knowledge many uncertainties remain, particularly during scale-up into industry relevant scales. Challenges occurring during scale-up can be inhomogeneities due to local accumulation of feed or base or merely poor mixing (Xu et al., 2018). Especially addition of titration agents like CO_2_ or Na_2_CO_3_ can lead to gradients and therefore inhibition of cellular growth and productivity (Xing et al., 2009; Sieblist et al., 2011). The result of these inhomogeneities can be gradients in pCO_2_ or pH influencing the cellular metabolism and hence the productivity of the process (Langheinrich and Nienow, 1999; Osman et al., 2001; Xing et al., 2009). In contrast, a shift to lactate consumption by the cells is considered as beneficial and can even occur when glucose is present in excess, but is not yet fully understood (Mulukutla et al., 2012). Since high lactate concentrations lower the pH and can downregulate certain enzymes it is favorable to avoid an accumulation of lactate (Leite et al., 2011). Effect of these parameters on intracellular metabolic fluxes and the energy household was seldom published although these factors are known to be crucial for the outcome of the process (Russell, 2007; Dickson, 2014; Huang et al., 2017).

In this study, the influence of the parameters pH and pCO_2_ on a fed-batch production process was investigated. Regarding pCO_2_, a stimulus effect in early stages and its impact on the further cellular state was studied. Secondly, a slow pH shift downwards should give an insight into metabolic adjustments made due to sinking pH, whereas other negative side effects of base addition like rising osmolality were suppressed. On the basis of extracellular rates, intracellular metabolomics and flux balance analysis the process parameters could be linked to specific metabolic states and their corresponding production kinetics. The found connections can be used for process parameter adjustments to lead cells into a more efficient state or to identify inhibiting influences of gradients occurring after scale-up.

## Materials and Methods

### Cell Line, Seed Train, and Bioreactor Cultivation

The CHO cell line 2-09 producing the monoclonal antibody type IgG1 was provided by the industrial partner Boehringer Ingelheim and cultivated in chemically defined basal and feed media. After thawing the cryo culture, the cells were expanded in a seed train consisting of a sequence of six shake flasks (Corning, USA) ranging from 125 to 1,000 mL before seeding the bioreactor. The humidified incubator (Infors HT, Switzerland) was set to 37°C, 5% CO_2_ and 120 RPM. Bioreactor cultivations in fed-batch mode were performed in a 4-fold parallel bioreactor system DS1500ODSS (DASGIP, Germany) with a starting volume of 1.0 L and an initial cell density of 0.7 × 10^6^ cells/mL. The feed was added continuously starting at 24 h of process time. In case of glucose concentrations falling below 2 g/L a concentrated glucose solution was added as a bolus to re-install glucose levels of 4 g/L. Setpoints of dissolved oxygen and temperature were always fixed at 60% and 36.5°C, respectively. The pH setpoint of 6.95 in the first 48 h and 6.80 in the remaining process time was controlled using CO_2_ as acid and 1 M Na_2_CO_3_ as base. The deadband range of the pH controller was 0.05. Increased addition of CO_2_ was mainly necessary in the early stages of the cultivation due to the initial medium pH of 7.2 and because cell densities were still low. During the course of cultivation, lactate formation increased and amino acid consumption reduced which was reflected by changing pH control shifting to a minimum of 3% CO_2_ overlay and Na_2_CO_3_ addition. In addition to the reference process (REF) two different settings were performed in which the influence of the two titrating agents should be investigated in regard to pH and pCO_2_. The lower threshold for CO_2_ gassing was set to 3% of the inlet gassing for all processes, while the upper boundary was set to 15% for the process testing the influence of CO_2_ (COP) and 10% for the other processes. Additionally, for COP the proportional factor of the controller was increased and reset time decreased to ensure a higher pCO_2_. For the third process type the use of base was eliminated completely resulting in a slow pH shift downwards (NOB). Each of the three setting was performed in triplicates.

### Sampling and Sample Preparation

Sampling for cell density and extracellular measurements was done twice a day whilst intracellular metabolomics samples were taken four times in total in different cultivation phases. For extracellular sampling the sample port was flushed with 4 mL of cell suspension before taking 4 mL sample volume. To prevent CO_2_ gassing out 100 μL were separated twice and each diluted immediately 1:20 in 1,900 μL 0.01 M KOH. They were frozen at −20°C and used for the quantification of total inorganic carbon using Multi N/C 2100s (Analytik Jena, Germany) as described in Buchholz et al. (2014) after thawing. Additional 100 μL of the original sample were used to determine the cell density with a Cedex XS cell counter (Roche, Germany). The remaining sample volume was centrifuged at 800 g and 4°C for 5 min. Sixty microliter of the supernatant were used to determine triplicates of glucose and lactate concentrations using a Labotrace automatic analyzer (TraceAnalytics, Germany). The remaining supernatant was frozen at −70°C and used for antibody titer and amino acid measurements after thawing. The determination of intracellular metabolite concentrations followed the fast filtration protocol of Matuszczyk et al. (2015) with slight changes. Each experimental setting was replicated three times (bioreactor cultivations in triplicates) and each sample analyzed in triplicates. Samples were taken after 33 h (early growth phase, phase 1), 82 h (middle growth phase, phase 2), 154 h (early stationary phase, phase 3), and 238 h (early decline phase, phase 4) of process time. Before intracellular sampling cell densities were determined to ensure sampling of 30 × 10^6^ cells. The fast filtration protocol of Matuszczyk et al. (2015) was performed by using glass fiber filter A/D with a pore size of 3 μm (Pall, USA) applying 30 mbar vacuum. Then the cells were quenched with ice cold washing buffer adjusted to the pH and osmolality at each process time which was determined in previous experiments. The filters were shifted into sample cups and then frozen in liquid nitrogen and stored at −70°C. Metabolite extraction followed the procedure of Pfizenmaier et al. (2015) applying chloroform and methanol. The feed rates were reduced to compensate the volume drain for sampling.

### Extracellular Analytics

Osmolality was analyzed via freezing point depression with an Osmomat 030 (Gonotec, Germany). Antibody titers were determined by enzyme-linked immunosorbent assay (ELISA) as described by Pfizenmaier et al. (2015). Reversed phase HPLC for the detection of amino acids used Agilent 1200 (Agilent, USA) according to Buchholz et al. (2013). L-Ornithine was used as an internal standard.

### Intracellular Analytics

Concentrations of intracellular adenosine phosphates (AXP) were determined following the HPLC protocol of Pfizenmaier et al. (2016). Intermediates of glycolysis and tricarboxylic acid (TCA) cycle were quantified using Agilent 1200 HPLC combined with Agilent 6410B triple quadrupole mass spectrometer (Agilent, USA). The liquid chromatography isotope dilution mass spectrometry (LC-IDMS) method (Teleki et al., 2015) using 13C labeled algae (Vielhauer et al., 2011) as internal standard was used to quantify intracellular pools of the non-labeled metabolites. Intracellular alpha-keto acids concentrations were quantified with an adapted method (Zimmermann et al., 2014) with derivatization steps due to the high reactivity of the compounds.

### Calculation of Specific Rates

For the calculation of the extracellular cell-specific rates the measured concentrations of metabolites as well as the cell densities were interpolated with steadily derivable functions using the Shape Language Modeling (SLM) tool in MATLAB Version 2013a (The Mathworks, USA). To avoid overfitting not more than four splines were used and only positive values were allowed similar to Wahrheit et al. (2014). Glucose concentrations were not fitted for the processes with necessary bolus additions since they showed intrinsic step functions.

### Flux Balance Analysis

Flux Balance Analysis made use of the models of Sou et al. (2015) and Carinhas et al. (2013) using the biomass composition of Selvarasu et al. (2012). For the use within the Cobra Toolbox in MATLAB the inclusion of exchange reactions for all metabolites between the two compartments cytosol and extracellular region was done. This was necessary to account for the systems biology markup language (SBML) model structure, which is required by the toolbox. To consider not only directly ATP producing pathways, reactions for oxidative phosphorylation were added. A P/O ratio of 2.5 for NADH and 1.5 for FADH_2_ was assumed, so that ATP production could be calculated out of the total produced NADH and FADH_2_. The number of intracellular reactions was 99. The additional number of exchange reactions was 114. The total number of metabolites in both compartments was 240. The objective function was chosen to be the maximization of ATP yielding reactions. Input parameters were the measured extracellular cell-specific rates for growth, antibody production, glucose, lactate, and amino acids. Constraints for the optimization were selected according to the error of these rates for the bioreactor cultivations in triplicates of each setting. Therefore, the upper and lower bounds were set as plus-minus the error of the rates at the time point of analysis. Flux Balance Analysis was done at the same time points as the intracellular sampling but neglecting the first time point at 33 h in the early growth phase since calculated rates were too erroneous due to low cell densities. The resulting flux error was calculated with a Monte Carlo sampling method (Thiele et al., 2005) included in the Cobra Toolbox. The solution space was sampled with 2.5 × 10^6^ randomly distributed points using 2,500 starting points for each analyzed time point.

## Results

### Influence of Process Parameters on Cell Density, Substrate, and Product Profiles

**Figure 1:**
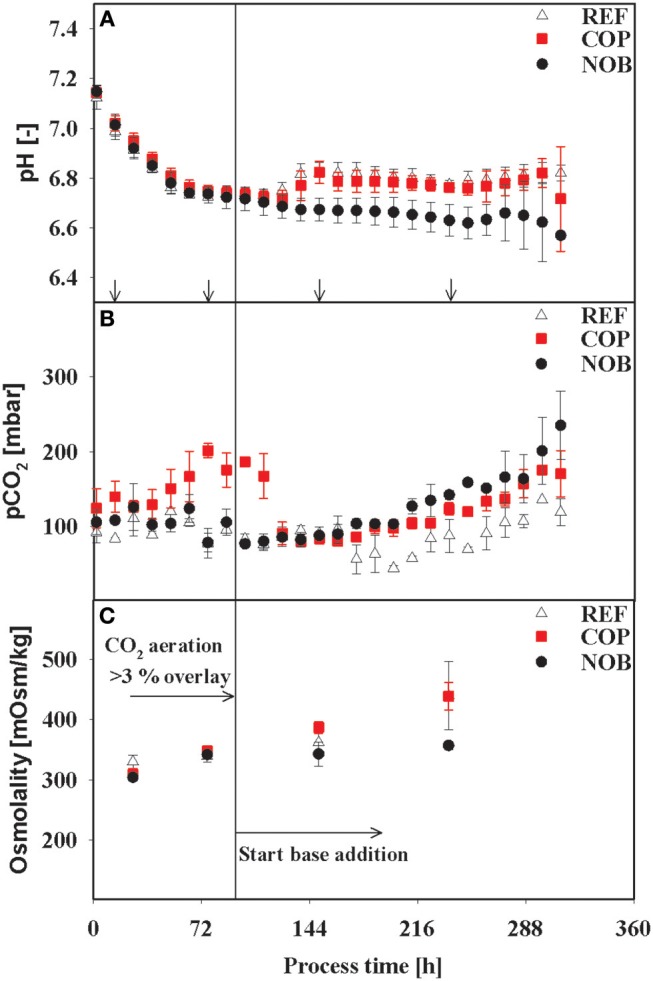
Courses of the process parameters pH **(A)**, pCO_2_ **(B)**, and Osmolality **(C)** for the three process settings. Osmolality was measured for each time point of intracellular sampling and flux balance analysis (no FBA performed for first time point). Arrows in **(A)** indicate the time points for intracellular samples, last three points also indicate time points of flux balance analysis. The vertical line in **(C)** marks the switch from CO_2_ aeration higher than the 3% overlay to base addition in the reference process. Errors were derived out of bioreactor cultivations in triplicates.

Bioreactor cultivations in triplicates of the setups REF (reference process), COP (increased CO_2_ impact), and NOB (no base titration) were performed as described above. Figure 1 gives an overview of the key parameters of the different process phases.

**Figure 2:**
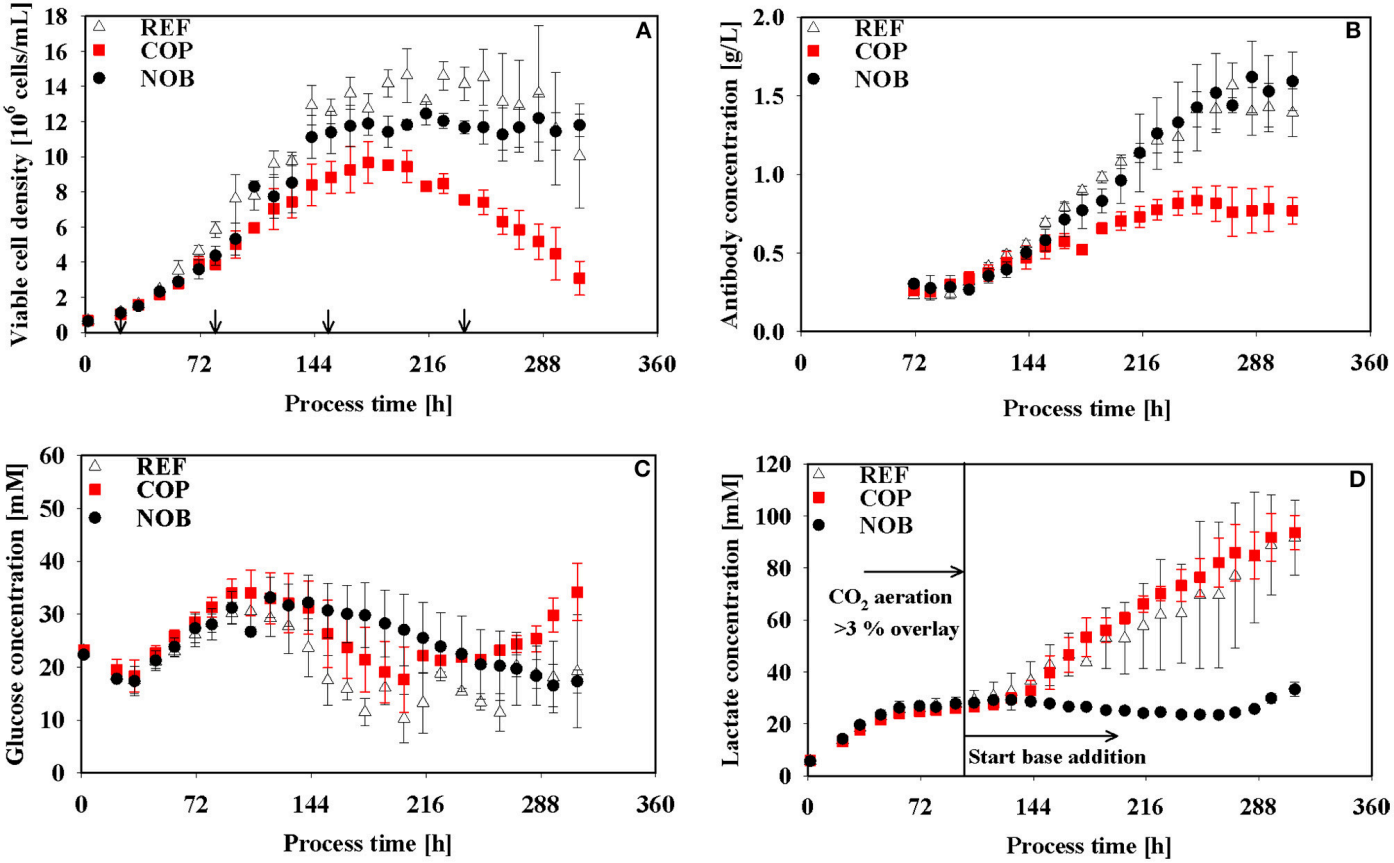
Viable cell density **(A)**, antibody titer **(B)**, glucose **(C)**, and lactate **(D)** concentrations over process time for the three different process settings. Arrows in **(A)** indicate the time points for intracellular samples, last three points also indicate time points of flux balance analysis. The vertical line in **(D)** marks the switch from CO_2_ aeration higher than the 3% overlay to base addition in the reference process. Error bars indicate the error of bioreactor cultivations in triplicates.

As expected, all cultivations were controlled at pH 6.8 after initial adaptation and diverged only after approximately 120 h when base addition was suppressed in setup NOB. The latter showed minimum pH of 6.60 whereas REF and COP remained stable within the controller's deadband of 0.05 of the respective process phase. The partial pressure of CO_2_ was elevated up to 200 mbar during the growth phase (83 h) of COP whilst values of REF and NOB ranged from 75 to 120 mbar. Until 120 h pCO_2_ values of all processes conformed to about 90 mbar, showed similar tendency for 48 h and diverged again >158 h with COP and NOB showing maximum pCO_2_ values at the very end of 170 and 220 mbar, respectively. Osmolality showed minor differences throughout the process, only in stationary phase the process without base addition (NOB) revealed significantly lower osmolalities of 357 mOsm/kg compared to the average values of REF and COP (440 mOsm/kg). REF and NOB showed similar courses of viable cell densities as depicted in Figure 2A. Maximum cell density was slightly higher for the reference process with 15 × 10^6^ cells/mL compared to 13 × 10^6^ cells/mL. In contrast, maximum cell density in COP reached only about 9 × 10^6^ cells/mL and cell densities fell earlier than for the other two processes. Highest final titer of 1.6 g/L was achieved in NOB which was slightly higher than in REF with 1.4 g/L and doubled the value of COP (Figure 2B). Frequent glucose addition was necessary for REF and COP reflecting sugar needs that exceeded the feed supply (Figure 2C). Strikingly, lactate concentrations (Figure 2D) steadily increased in REF and COP but remained stable on the low level of around 25 mmol/L > 72 h in NOB.

### Growth and Product Formation Kinetics

**Figure 3:**
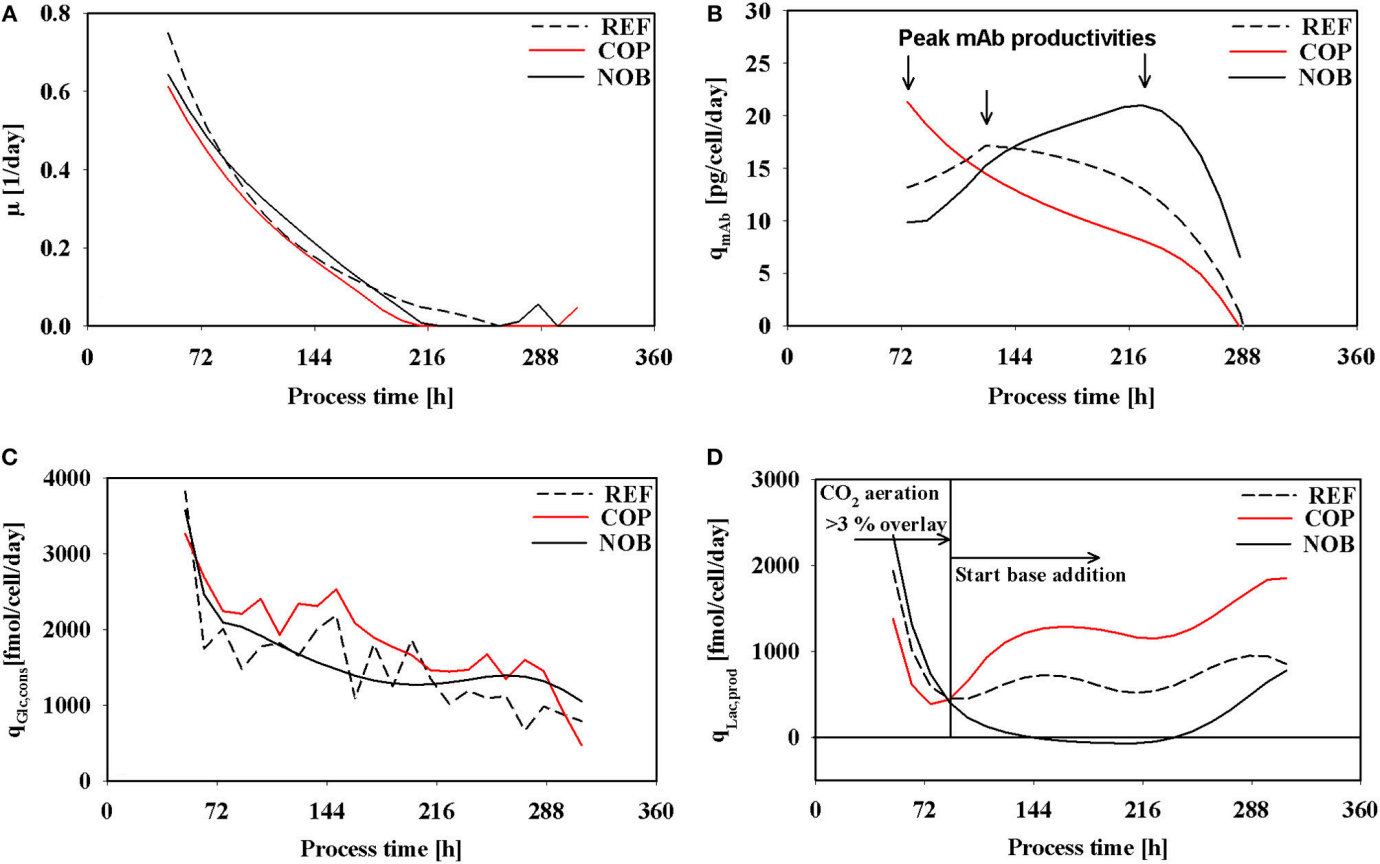
Specific rates of growth **(A)**, antibody production **(B)**, glucose consumption **(C)**, and lactate production **(D)** over process time for the three different process settings. The vertical line in **(D)** marks the switch from CO_2_ aeration higher than the 3% overlay to base addition in the reference process as described in Figure 1. Negative values in **(D)** indicate a switch from lactate production to lactate consumption.

Cell specific rates of growth, antibody production, glucose consumption, and lactate production are depicted in Figure 3. All growth rates (Figure 3A) indicate the same declining trend irrespective of the setting although maximum cell densities of COP were the lowest (Figure 2A). In REF growth continued until 264 h whereas in COP and NOB growth stopped after 216 h. The altered process settings resulted in different kinetics of productivity of the monoclonal antibody as can be seen in Figure 3B. REF and NOB depicted similarly increasing productivities until 120 h when productivity peaked at 18 pg/cell/day in REF. Notably, cell specific productivities declined in REF whereas in NOB they rose steadily to the maximum of 20 pg/cell/day (240 h). On contrast, q_mAb_ values in COP steadily declined from the initial value of 23 pg/cell/day when pCO_2_ was still elevated (Figure 1). Time courses of glucose uptake rates (Figure 3C) showed similar declining trends in all settings. However, COP revealed highest demands. A striking observation is shown in Figure 3D: Specific lactate production rates showed high similarities during the first 72 h but differed significantly after base addition. Whereas, lactate formation almost persisted in REF, COP showed strong rise and NOB even intermediary stop. As a result the lactate production rate was about 70% higher in COP compared to REF. In NOB, cells even switched from lactate production to consumption between 144 and 240 h (compare Supplementary Figures S1–S3 for carbon balances).

**Figure 4:**
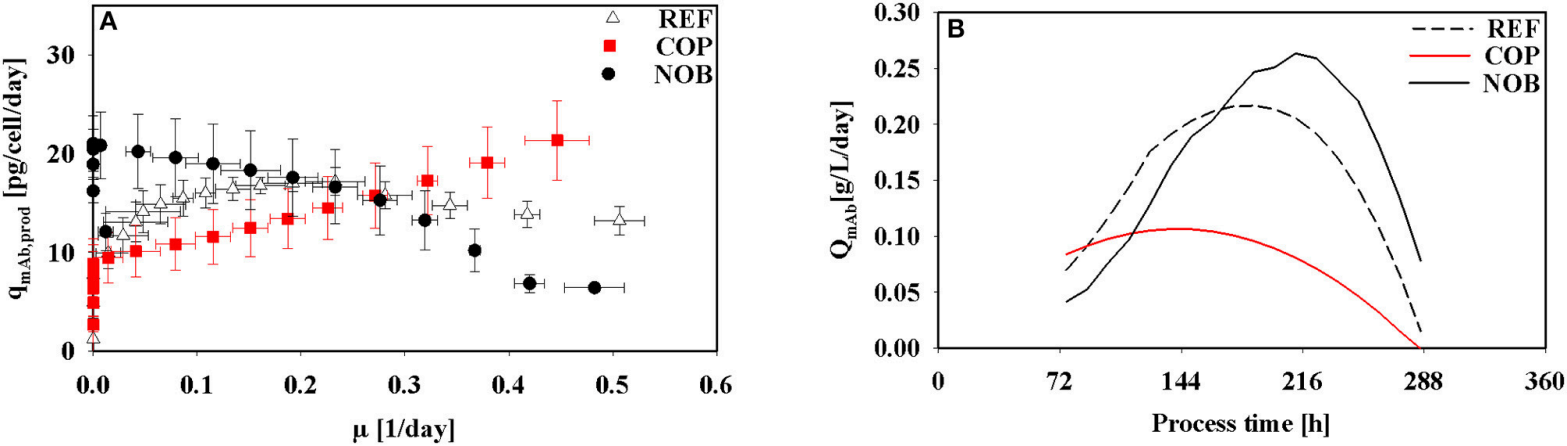
Kinetics of antibody production in dependence of growth rate **(A)** and volumetric productivity of the antibody over process time **(B)** for the three different process settings.

Interestingly, kinetics of cell specific antibody formation q_mAb_ as a function of μ (Figure 4A) revealed properties depending on the process setting. In REF highest productivities were observed in the operational window of moderate growth (0.2 1/d). On contrast, COP showed highest productivities linked to highest growth rates (0.4 1/d). Highest productivities in NOB were achieved at low growth rates approaching the stationary phase when maximum cell densities were present.

The volumetric productivity for all process types is depicted in Figure 4B. REF and NOB showed a rising trend toward the stationary phase due to increasing cell densities (Figure 2A). But since for REF the specific productivity (Figure 3B) started decreasing after 120 h its volumetric productivity peaked at 0.2 g/L/day after 192 h and therefore 36 h earlier than in NOB with a maximum value of 0.25 g/L/day. COP reached its peak value of 0.1 g/L/day after 144 h because of the lowest cell densities along with decreasing cell specific productivities from 72 h on.

### Flux Balance Analysis and Intracellular Metabolite Levels

**Figure 5:**
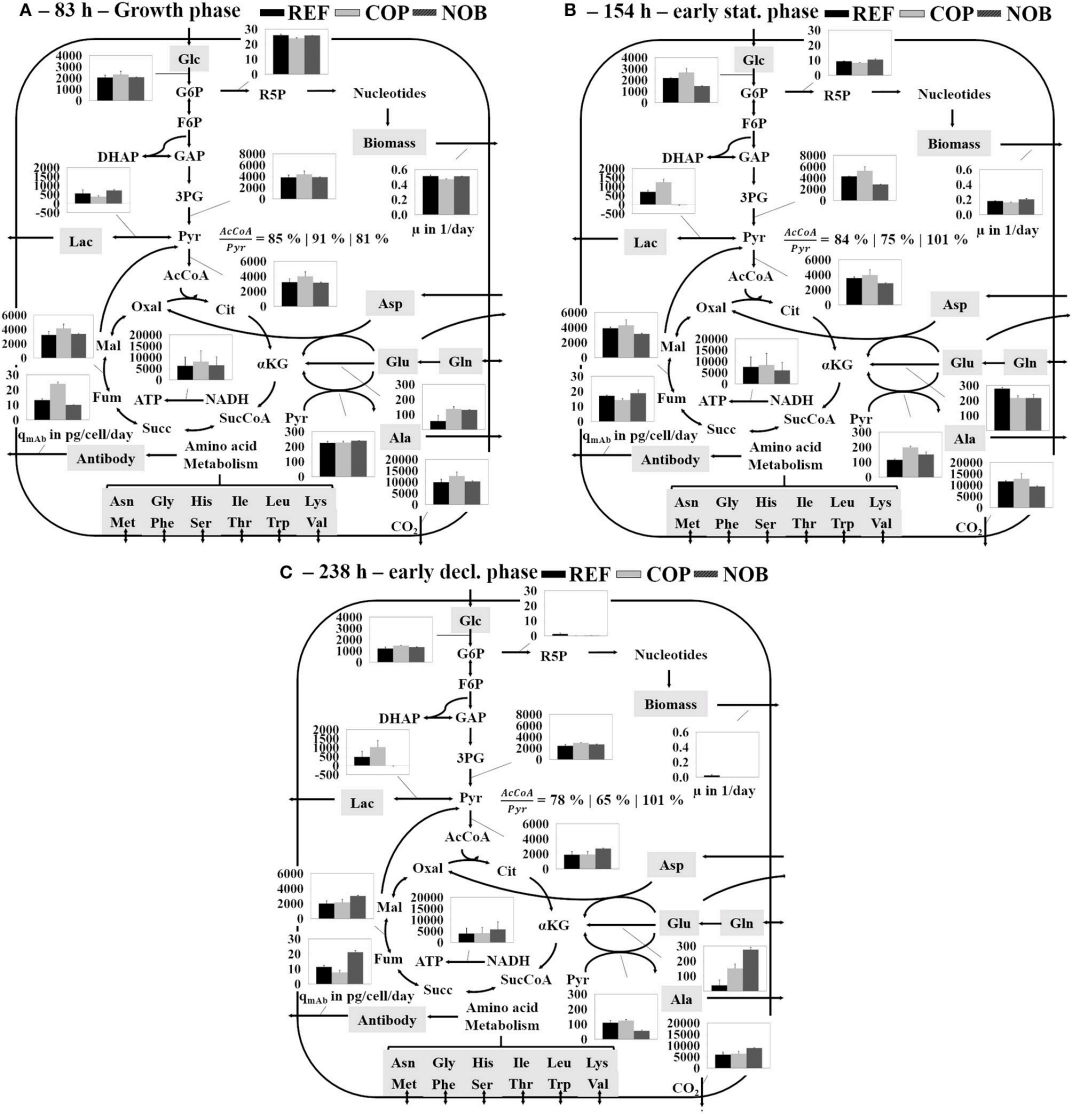
Simplified overview of the results of the flux balance analysis depicting the main carbon metabolism during growth phase **(A)**, early stationary phase **(B)**, and early decline phase **(C)** for all three process settings. The three bars of each flux belong to the according process shown in the top right. Fluxes are given in fmol/cell/day unless otherwise stated. Errors were determined through Monte Carlo sampling of the solution space of each the bioreactor cultivations in triplicates.

Flux balance analysis was performed for all settings for growth phase (83 h, phase 2), early stationary phase (154 h, phase 3) and early decline phase (238 h, phase 4; see Figure 5). In accordance to the extracellular rates shown in Figure 3 the glycolytic flux patterns of phase 2 (Figure 5A) were comparable for all three settings with only marginally increased fluxes in COP. Accordingly, the flux into TCA was slightly higher in COP as well as the drain into TCA from the pyruvate knot. The remaining pyruvate was converted to lactate (around 500 fmol/cell/day) and alanine (200 fmol/cell/day) for all three processes. At the beginning of the stationary phase (Figure 5B) fluxes through glycolysis and TCA remained constant in REF. The 20% increased conversion rate from 3PG to pyruvate in COP was solely used for the production of lactate. Accordingly, lactate dehydrogenase flux doubled to 1,200 fmol/cell/day, but flux into TCA persisted. In NOB, the glycolytic flux decreased by a third and lactate production stopped which resulted in constant TCA flux activity compared to the growth phase. Energy production via oxidative phosphorylation remained at the same level for all three processes. During phase 4 (Figure 5C) the flux through glycolysis was cut in half in REF and in COP whilst fluxes remained constant in NOB. Activity of pentose phosphate pathway apparently broke down in all processes. Interestingly, only NOB could preserve TCA fluxes even in phase 4. REF and COP revealed declines. In essence, this was the result of no lactate formation and reduced alanine formation from pyruvate.

**Figure 6:**
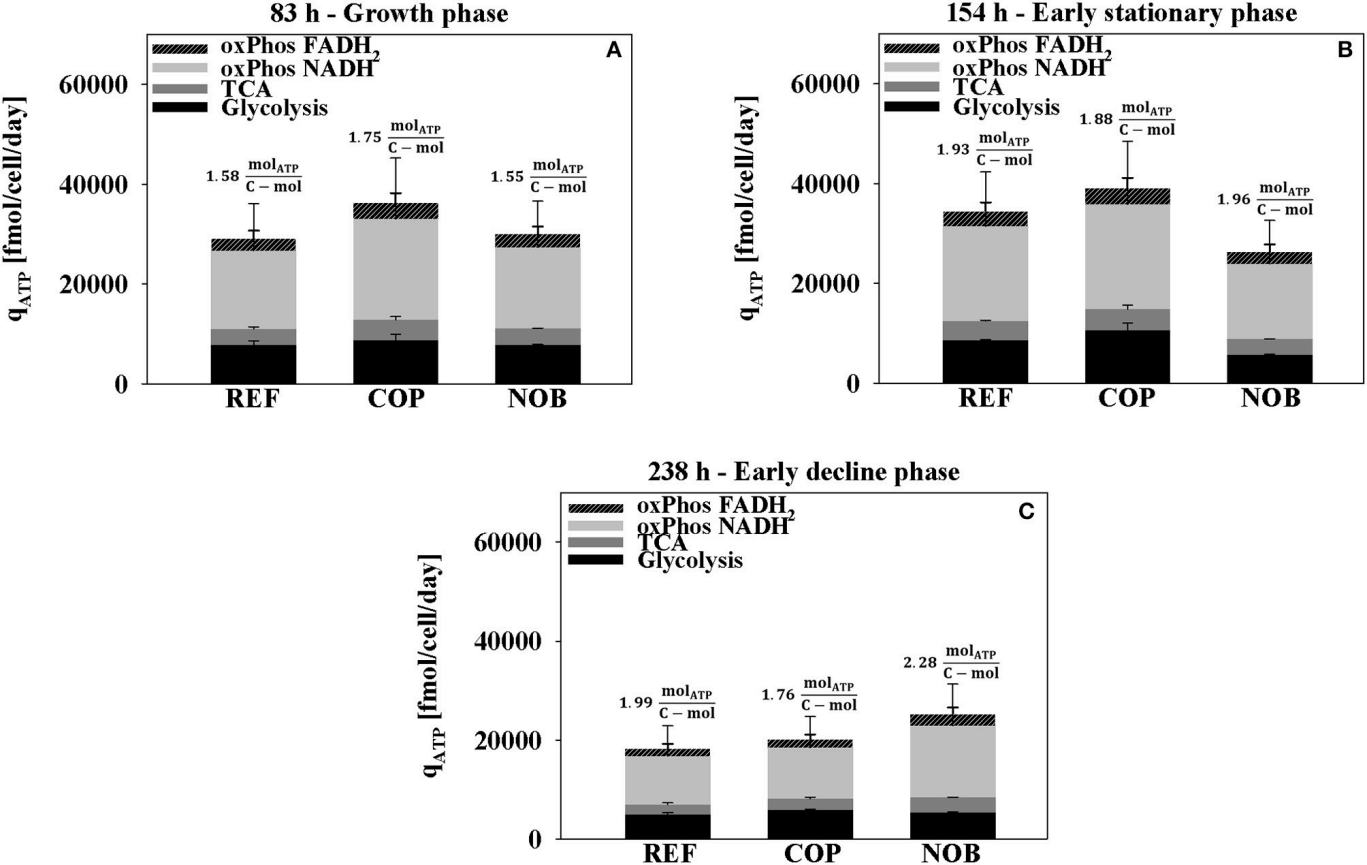
ATP production rate during growth phase **(A)**, early stationary phase **(B)**, and early decline phase **(C)** for the three process settings divided into the origin of ATP formation. Values for oxidative phosphorylation include NADH or FADH_2_ produced in both glycolysis and TCA. ATP directly from TCA originates from succinyl-CoA synthetase. Results were obtained from the flux balance analysis. Errors were determined through Monte Carlo sampling of the solution space of each the bioreactor cultivations in triplicates.

Figure 6 depicts that highest cell specific ATP production rates were found in phase 2 of COP. q_ATP_ increased by 10% in REF and in COP in the stationary phase (phase 3) whereas it decreased by 10% for NOB (Figure 6B). The ATP production yield Y_ATP, C_ was estimated as ATP generated per c-mol substrate consumed. The criterion turned out to be similar for all three processes with values ranging from 1.88 to 1.96 molATP/c-mol. Both, REF and COP showed halving of ATP formation in phase 4 (Figure 6C) when NOB could maintain the previous level of ~25,000 fmol/cell/day. The development was accompanied by severely increasing Y_ATP, C_ to 2.28 molATP/c-mol. Throughout the cultivation the ratio between the sources of ATP generation remained similar. The oxidative phosphorylation of total NADH and FADH_2_ contributed about 65% to the energy produced. ATP out of succinyl-CoA synthetase in the TCA accounted for 10% and ATP out of glycolysis for 25%.

**Figure 7:**
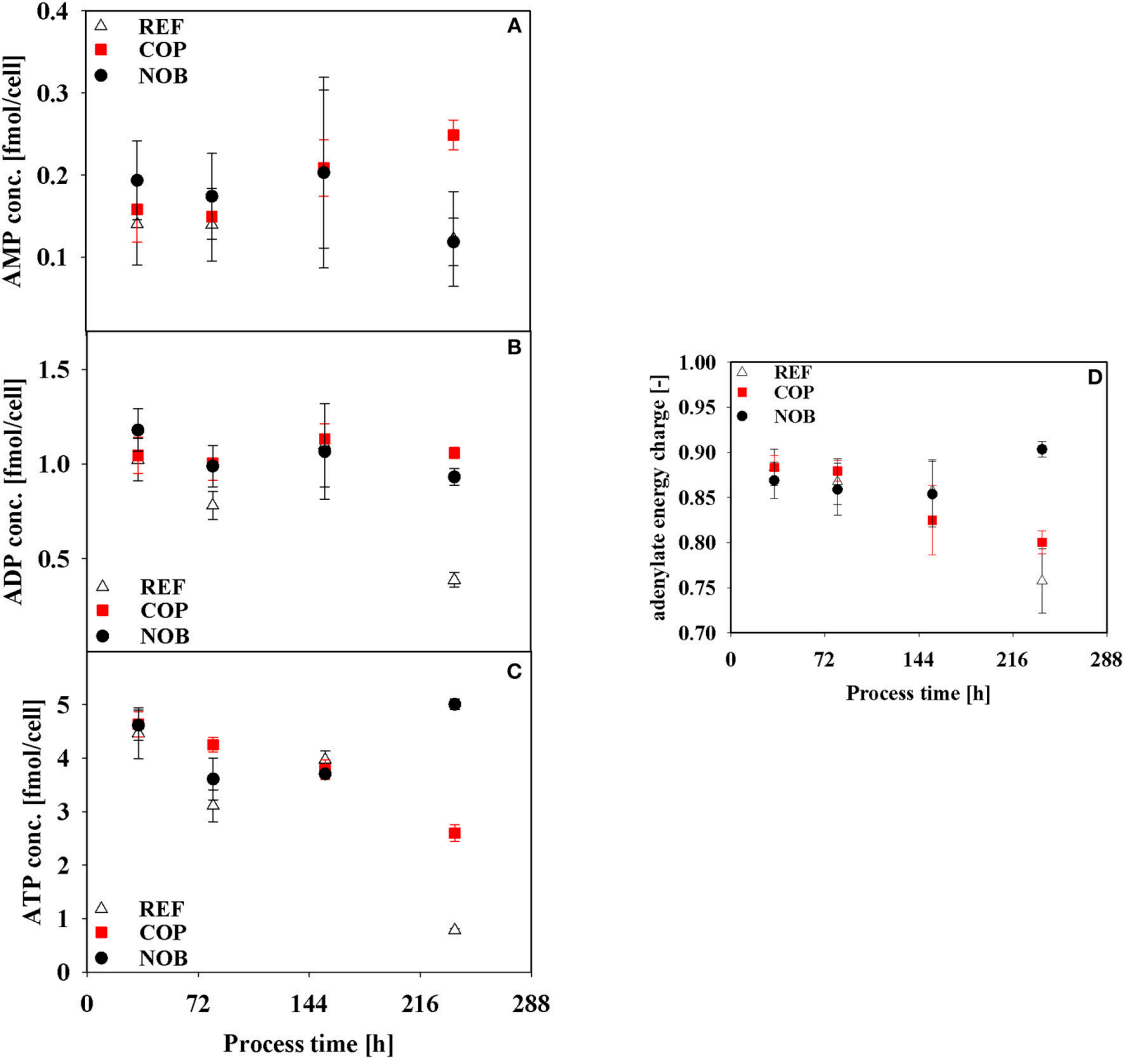
Intracellular concentrations of AMP **(A)**, ADP **(B)**, ATP **(C)**, and adenylate energy charge **(D)** during the three different processes. Error bars indicate the error of measurement of samples in triplicates.

Results of intracellular measurements of nucleotides as well as the resulting adenylate energy charge (AEC) are depicted in Figure 7. Major differences between the settings could be found in phase 4 when AEC was highest for NOB with a value of 0.90 whilst the other processes revealed an ever declining trend resulting in AECs of 0.76 and 0.80.

**Figure 8:**
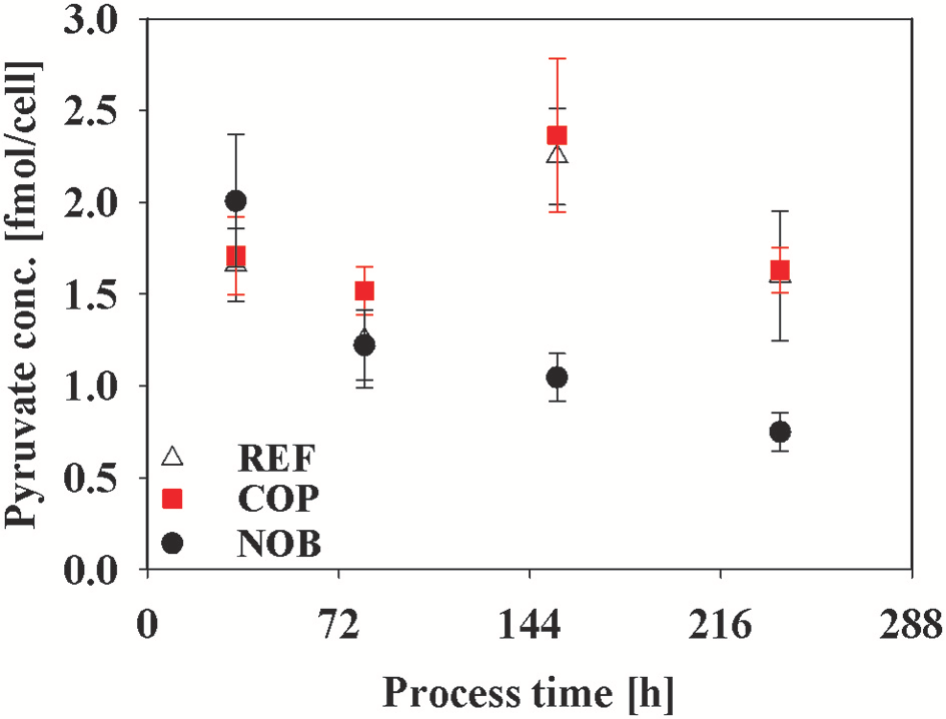
Intracellular concentration of pyruvate during the three different processes. Error bars indicate the error of measurement of samples in triplicates.

Intracellular metabolite concentrations of Glycolysis and TCA did not show large deviations in between the three process types (data not shown). Only intracellular pyruvate concentrations in Figure 8 showed a steady decline for NOB whereas the other two processes reached maximum pool concentrations of 2.20 fmol/cell in phase 3 and even showed doubled pool sizes at the end of the processes compared to NOB.

## Discussion

### Impact of pH and Elevated CO_2_ Levels on Growth and By-Product Formation

As depicted in Figure 1, pH, pCO_2_, and osmolality courses differed between the three settings. NOB revealed low pH reflecting eliminated base addition after entering the stationary phase. COP had elevated pCO_2_ levels during growth phase. Interestingly, only NOB disclosed stop of lactate production after base feeding had been prevented. The link between low pH and initiation of lactate consumption was already reported by Ivarsson et al. (2015), Liste-Calleja et al. (2015), and Zalai et al. (2015). Typically step-wise pH down shifts were investigated. Furthermore, high pH settings were found to increase q_Lac_ (Yoon et al., 2005).

Lao and Toth (1997) published cell line specific threshold values of 60 mM lactate indicating the start of detrimental effects on growth rates. This level was reached in the early stationary phase of REF and COP (Figure 2D) consequently leading to the decrease of viable cell density earlier in REF and COP than in NOB. Moreover, increased lactate production caused equal rise of base addition reflected by elevated osmolalities in REF and COP compared to NOB (Figures 1, **2D**). High osmolalities were known to cause reduction of growth and viable cell densities (Lin et al., 1999) and an increase in lactate formation (Xu et al., 2018). Furthermore, Pfizenmaier et al. (2015) outlined that cells were increasingly arrested in G1 phase under hyperosmotic conditions finally improving cell specific productivities of recombinant mAb production. However, considering osmolalities >400 mOsm/kg as hyperosmotic (Pfizenmaier et al., 2015) such stress conditions were only reached in the decline phase >238 h (Figure 1) which rules out any further impact before.

### The Interaction of pH and Elevated CO_2_ Levels With mAb Production

The impact of elevated pCO_2_ pressures on cell specific productivities is not fully elucidated yet. Partial CO_2_ pressures of 290 mbar were found to deteriorate average specific productivities (Gray et al., 1996; Kimura and Miller, 1996; Zanghi et al., 1999; Mostafa and Gu, 2003; Goudar et al., 2007) and to rise lactate production (Darja et al., 2016; Brunner et al., 2017, 2018). On contrast, some studies did not find any significant effect on cell specific productivities but only on growth (DeZengotita et al., 2002; Takuma et al., 2003). All studies have in common that high pCO_2_ values were installed throughout the complete process thereby preventing time-resolved analysis of interactions between pCO_2_ levels, process dynamics and cell specific productivities. By trend, elevated pCO_2_ values were found to reduce q_mAb_. However, particularities of individual cell lines and product formation kinetics need to be analyzed for each individual case.

Exploiting the experimental settings for REF, COP, and NOB, the impact of different pCO_2_ and pH levels on q_mAb_ courses can be deciphered for the process periods revealing two outstanding observations:

- *Cell specific productivities were high despite high pCO*_2_ *values*: COP showed maximum q_mAb_ in early stages when CO_2_ stress was present (Figure 3B) and growth rates were similar in all experimental settings. The observation is in agreement with proteomic studies of Darja et al. (2016) linking elevated pCO_2_ with the amplification of the stress protein GRP78 which in turn was correlated with increased productivities in CHO protein producers by Pieper et al. (2017) and Nishimiya et al. (2013).
- *NOB production kinetics are uncoupled from growth:* In COP, q_mAb_ kinetics were tightly coupled to cellular growth (Figure 4A). REF showed similar trends, however without the initial benefits of (i). In both settings cell specific productivities and growth rates steadily decreased after about 120 h until the end. Strikingly, NOB kinetics were significantly different. Although growth rates reduced parallel to REF and COP, cell specific productivities did not. On contrast, q_mAb_ kept raising until 216 h increasing values by almost 20% (Figure 2D). The dynamics coincided with slightly falling pH in NOB. The observation is in accordance with other studies outlining either beneficial (Ivarsson et al., 2015; Brunner et al., 2018) or no significant (Yoon et al., 2005; Trummer et al., 2006; Zalai et al., 2015) impact of pH downshifts on cell specific productivity. The finding also conforms to studies of Pfizenmaier et al. (2016) outlining that cell specific productivities can be increased under non-growing conditions, presumably because of elevated supply of ATP. In NOB, the effects led to maximum specific productivities of 20 pmol/cell/day which were slightly higher than in REF. However, most desirable is a high productivity coinciding with high viable cell densities to reach the highest volumetric output. This could be achieved for NOB due to specific productivity being maintained at a high level (Figure 4B). COP on the contrary peaked in specific productivity during growth phase where cell densities were still low and therefore the titer was severely decreased. Interesting enough, maximum cell specific productivities in REF, COP, and NOB coincided with minimum lactate formation. Therefore, intracellular flux distributions should be used to decipher the metabolic adjustments made due to changing process conditions.

### Metabolic and Energetic Adjustments Due to Shifted pH and Elevated pCO_2_

Only recently more studies investigated the influence of process parameters on a more detailed level via metabolic flux analysis for pCO_2_ effects (Brunner et al., 2018) and flux balance analysis for pH effects (Ivarsson et al., 2015). However, the connection between changes of production kinetics and energetic state over distinct process phases has not been unraveled yet.

The levels of glucose uptake and lactate production could only be correlated for the early stationary phase comparing the three process types and not throughout the process as described before (Zalai et al., 2015; Konakovsky et al., 2016; Brunner et al., 2018). Nevertheless, COP showed the highest glycolytic fluxes throughout the process (Figure 5). Lower fluxes in NOB were compensated through slight lactate uptake so that TCA fluxes were similar to these of the other processes. This effect was shown by Ivarsson et al. (2015) during a batch process with pH 6.8 compared to a reference process with a pH of 7.2. Possible factors inducing lactate uptake were hypothesized to be pH dependent glycolytic enzymes or a cellular measure to increase extracellular pH again (Ozturk et al., 1992). Higher levels of intracellular pyruvate promoted overflow metabolism for REF and COP in terms of increased lactate production (Figure 8). Lower pH in NOB coincided with decreased pyruvate levels and simultaneous lactate uptake >144 h. Luo et al. (2012) showed similar results for relative intensities of intracellular pyruvate when comparing different cell lines.

The analysis of intracellular fluxes revealed furthermore that a high efficiency at the pyruvate branch toward TCA and therefore low lactate production were coinciding with high specific productivities (Luo et al., 2012; Templeton et al., 2013). The CO_2_ stressed environment in the early stages of COP revealed a remarkable energy producing state with low lactate production. The elevated ATP production was ongoing until early stationary phase at 154 h although pCO_2_ went back to reference values around 24 h before. However, intracellular ATP concentrations and productivity had already begun diminishing while lactate formation was increasing. Therefore stress conditions of elevated pCO_2_ might lead to a higher ATP usage due to the decrease in intracellular pH and therefore higher maintenance demands (Ozturk et al., 1992). DeZengotita et al. (2002) showed that increased partial pressures of CO_2_ led to a reduction in pH_i_ of around 0.2 since CO_2_ as a non-polar molecule can easily diffuse through the cellular membrane. To keep intracellular pH from falling further, ATP consuming transporters are known which continuously extrude protons out of the cell (Roos and Boron, 1981; Casey et al., 2010). The more CO_2_ enters the cells, the less ATP can be used for energy demanding protein synthesis which would explain the rapidly falling specific productivities.

On the contrary the amount of ATP produced per cell stayed on a constant lower level for NOB. A smaller q_ATP_ was shown in a batch with lowered pH of 6.8 along with higher average specific productivities compared to a reference with pH 7.2 (Ivarsson et al., 2015) and for cells after the switch to lactate consumption in a batch with depleted glucose (Martínez et al., 2013). Contrary to the other two processes NOB could maintain a high intracellular AEC and stable intracellular ATP concentrations along with constant q_ATP_ and increasing q_mAb_, even toward the decline phase. Pfizenmaier et al. (2015) also showed an increased productivity along with higher intracellular ATP concentrations under non-growing conditions for cells under osmotic stress. The same could be observed for NOB with a shift toward non-growth coupled product formation. The shift downwards in pH might be not as severe as the applied CO_2_ stress in COP. Hence the cells can apply countermeasures toward a physiological pH_i_ (Wu et al., 1993; Brunner et al., 2017) without having to spend as much ATP.

Our results indicate that there is a strong connection between the process parameters CO_2_ and pH on the one side and the production kinetics and the cellular energetic efficiency on the other side. The NOB approach ensures steady production conditions which may be of particular importance when the process should be scaled up in industrial settings. CO_2_ and pH can be crucial parameters creating non-wanted population inhomogeneities. Following the NOB setting the problem should be reduced which in turn should increase the chances of successful scale-up.

A short CO_2_ stimulus—for example due to an unoptimized controller or poor mixing (Xu et al., 2018)—in the early stages of a process can have long-term detrimental effects even when pCO_2_ is back to reference values. Although specific productivity was shortly increased under stress conditions during early growth phase, the altered kinetics of productivity and lactate production combined with decreasing ATP levels revealed a fatal development for the process. A slow pH shift downwards on the other side turned out to be advantageous for the ongoing process. Substrate uptake was kept low and lactate was partly consumed. A shift toward non-growth coupled production was observed until highest cell densities were reached. Constantly high adenylate energy charges throughout the process together with persistent ATP production rates proved to be beneficial for maintaining high productivities.

## Author Contributions

MB performed the cultivations, extracellular analytics and modeling, designed the study, and prepared the manuscript. MB, LJ, and AT performed the intracellular metabolomics. JB performed critical revision of the data. RT designed the study and prepared the manuscript.

### Conflict of Interest Statement

JB was employed by company Boehringer Ingelheim Pharma GmbH and Co. KG. The remaining authors declare that the research was conducted in the absence of any commercial or financial relationships that could be construed as a potential conflict of interest.

## Abbreviations

REF: reference process
COP: CO_2_ stressed process
NOB: no base titration process.
