# Supplementary material for: The Less the Better: How Suppressed Base Addition Boosts Production of Monoclonal Antibodies With Chinese Hamster Ovary Cells
Source: Front Bioeng Biotechnol. 2019 Apr 11;7:76. doi: 10.3389/fbioe.2019.00076 (PMC6470187; doi:10.3389/fbioe.2019.00076)
Supplement: Supplementary file 1 [file Table_1.DOCX]

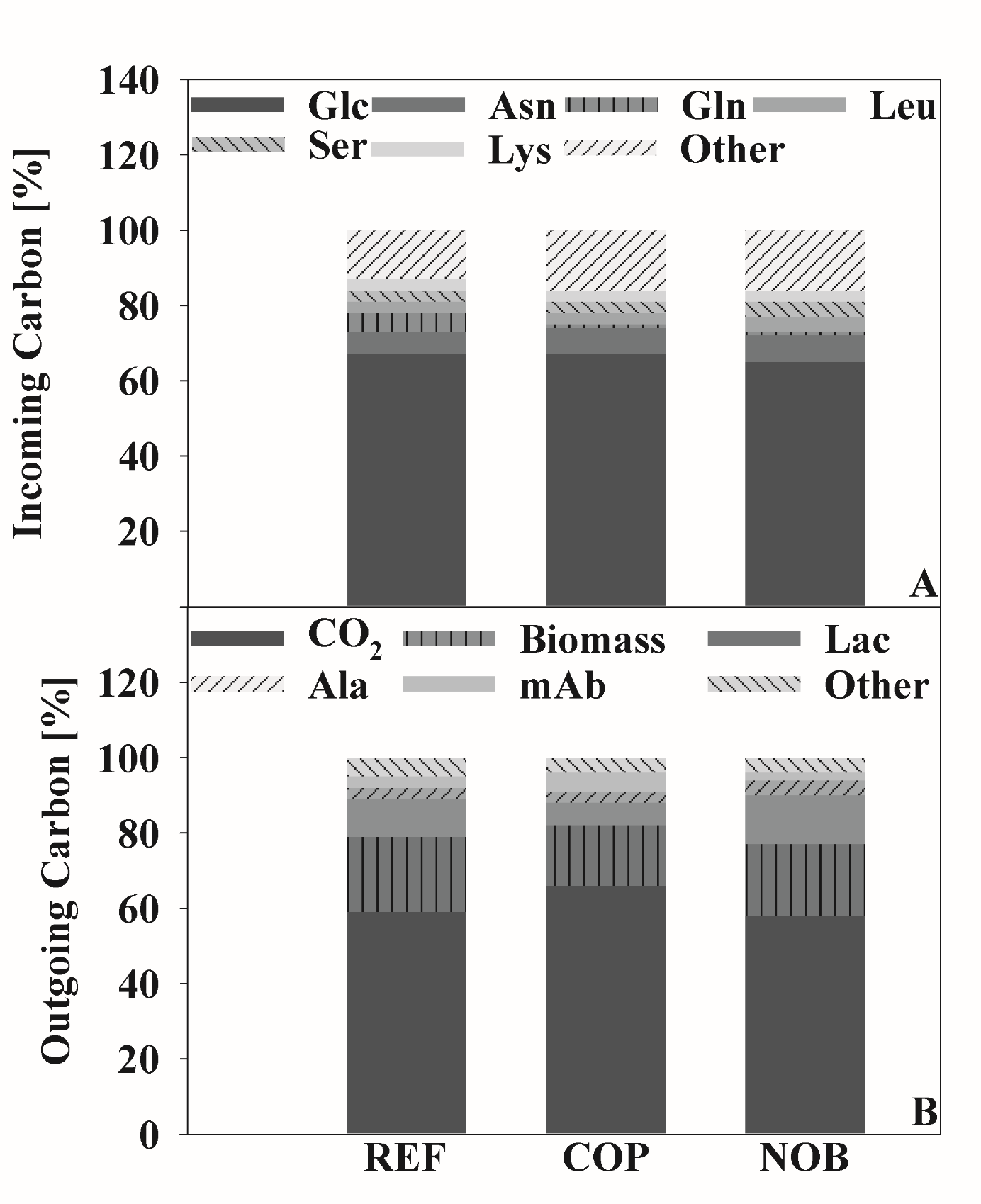


**FIGURE S.1** | Carbon balances of incoming substrates and outgoing products for the three process settings during growth phase (83 h). Indicated are the fractional contributions for incoming (A) and outgoing (B) carbon. Amino acids with minor contributions are lumped into *other*. Balances were calculated from the results of the flux balance analysis.


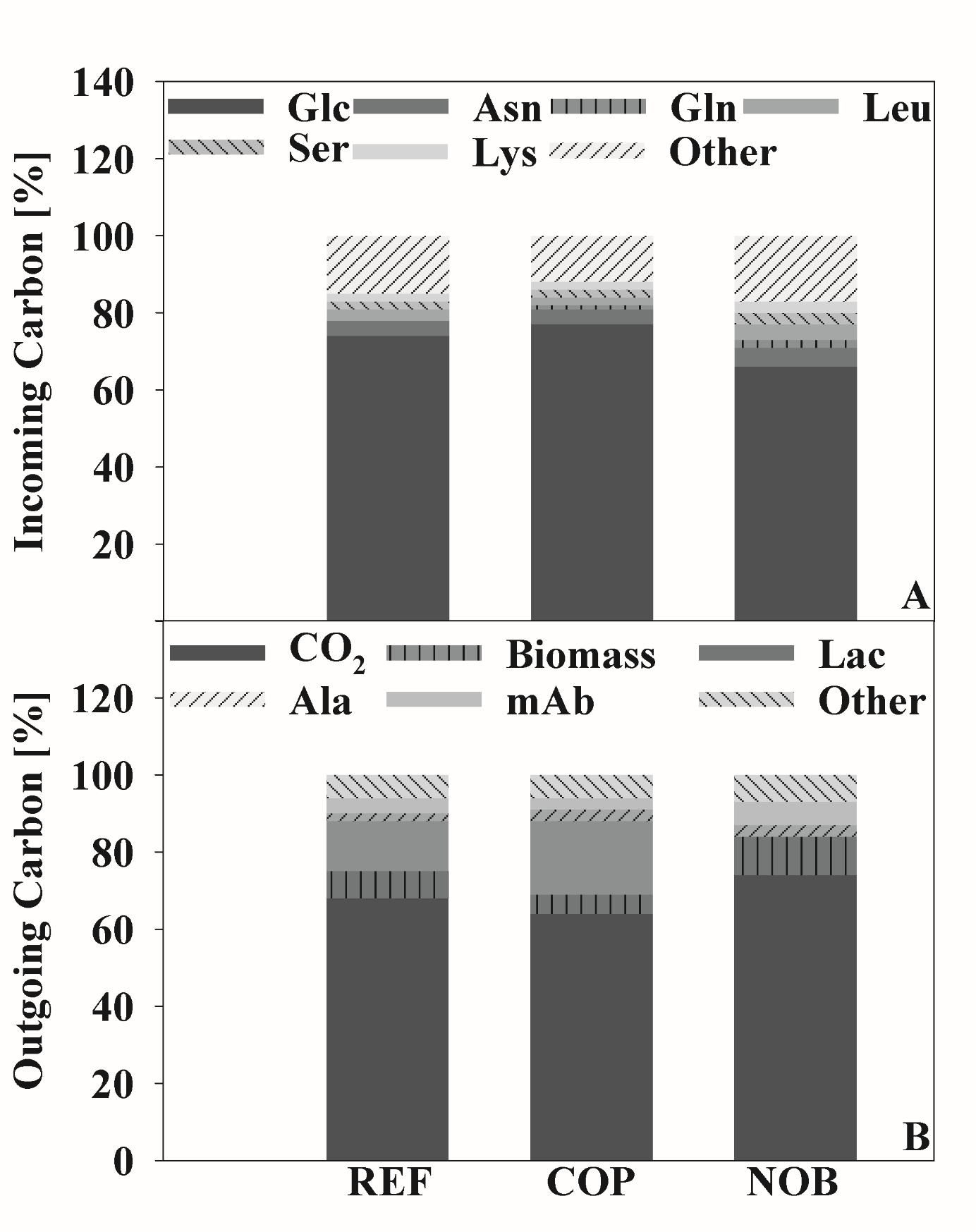


**FIGURE S.2** | Carbon balances of incoming substrates and outgoing products for the three process settings during early stationary phase (154 h). Indicated are the fractional contributions for incoming (A) and outgoing (B) carbon. Amino acids with minor contributions are lumped into *other*. Balances were calculated from the results of the flux balance analysis.


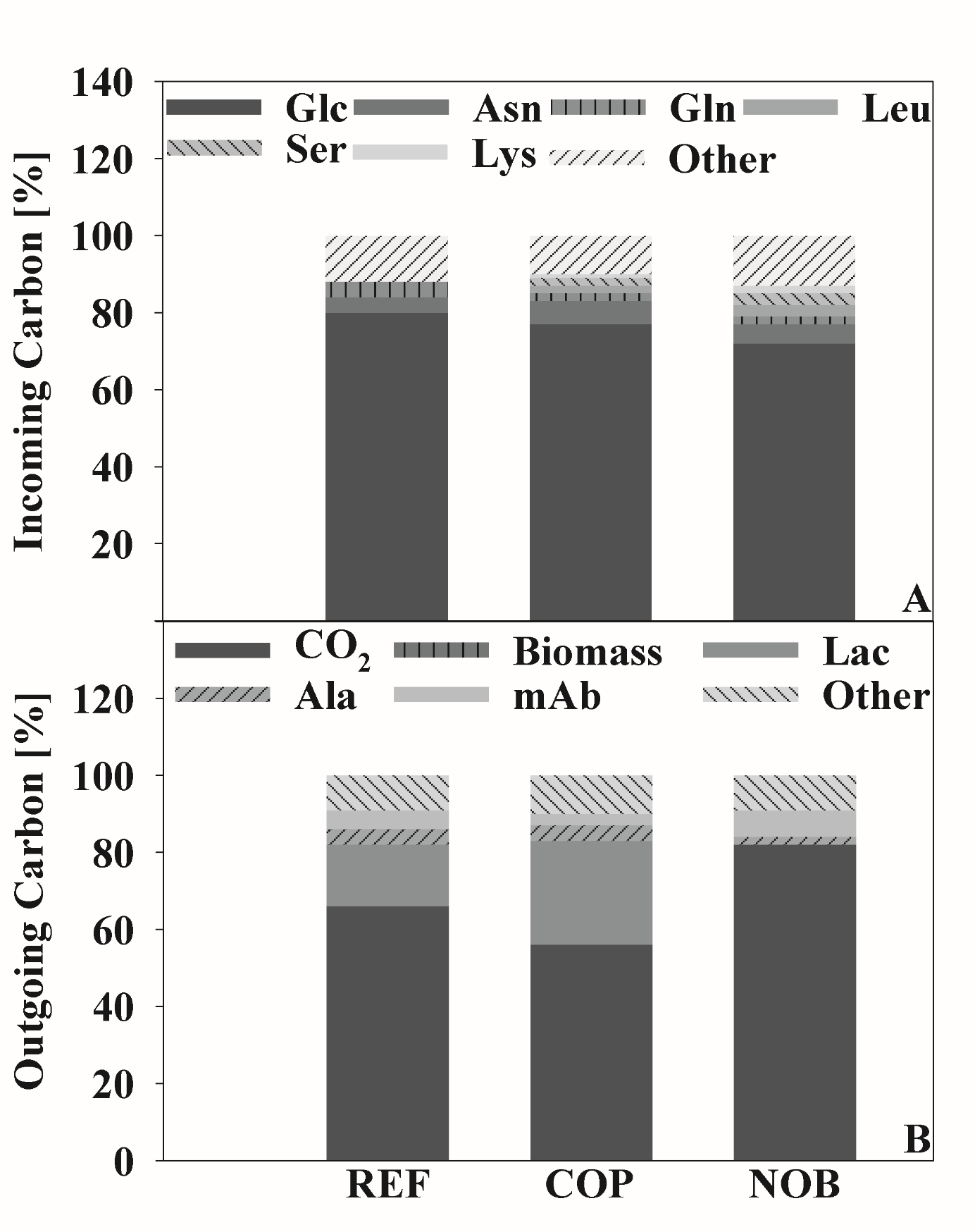


**FIGURE S.3** | Carbon balances of incoming substrates and outgoing products for the three process settings during early decline phase (238 h). Indicated are the fractional contributions for incoming (A) and outgoing (B) carbon. Amino acids with minor contributions are lumped into *other*. Balances were calculated from the results of the flux balance analysis.
